# The Need for Co-Creation of Care with Multi-Morbidity Patients—A Longitudinal Perspective

**DOI:** 10.3390/ijerph17093201

**Published:** 2020-05-05

**Authors:** Sanne J. Kuipers, Anna P. Nieboer, Jane M. Cramm

**Affiliations:** Department of Socio-Medical Sciences, Erasmus School of Health Policy & Management, Erasmus University Rotterdam, 3000 DR Rotterdam, The Netherlands; nieboer@eshpm.eur.nl (A.P.N.); cramm@eshpm.eur.nl (J.M.C.)

**Keywords:** co-creation of care, primary care, multi-morbidity, social well-being, physical well-being, satisfaction with care

## Abstract

Background: Primary care delivery for multimorbid patients is complex, due to single disease–oriented guidelines, complex care needs, time constraints and the involvement of multiple healthcare professionals. Co-creation of care, based on the quality of communication and relationships between healthcare professionals and patients, may therefore be valuable. This longitudinal study investigates the relationships of co-creation of care to physical and social well-being and satisfaction with care among multimorbid patients in primary care. Methods: In 2017 and 2018, longitudinal surveys were conducted among multimorbid patients from seven primary care practices in Noord-Brabant, the Netherlands (*n* = 138, age = 73.50 ± 9.99). Paired sample t-tests and multivariate regression analyses were performed. (3) Results: Co-creation of care improved significantly over time (*t* = 2.25, *p* = 0.026), as did social well-being (*t* = 2.31, *p* = 0.022) and physical well-being (*t* = 2.72, *p* = 0.007) but not satisfaction with care (t = 0.18, *p* = 0.858). Improvements in co-creation of care from T0 to T1 were associated with social well-being (B = 0.157, *p* = 0.002), physical well-being (B = 0.216, *p* = 0.000) and satisfaction with care (B = 0.240, *p* = 0.000). (4) Conclusions: Thus, investment in co-creation of care by primary care practices may lead to better outcomes for multimorbid patients.

## 1. Introduction

The global prevalence of multi-morbidity is increasing [1]. As multi-morbidity is associated with age, its prevalence is expected to increase even further in the near future due to populational ageing [2,3]. Multi-morbidity is often described as the co-existence of two or more chronic conditions in one patient [4]. It has been associated with poorer health outcomes, such as reduced functional capacity and quality of life, as well as increased healthcare use [5,6,7,8,9,10].

In the Netherlands, most care delivery for patients with multi-morbidity is managed by general practitioners (GPs) in the primary care setting [11]. The management of care delivery for this patient population is complex; it is costly and difficult due to patients’ complex care needs [12], and the single-disease orientation of many guidelines and protocols results in uncertainty about what best care is for patients with multiple diseases [13,14,15]. Thus, patients with multi-morbidity receive care that is often fragmented and not tailored to their needs, which may result in irrelevant or potentially unsafe treatment [15,16]. Care management for patients with multi-morbidity is also complex because time constraints often result in suboptimal approaches to care delivery [15]. Most consultations last 10–20 min, and most of this time is used to efficiently discuss all medically related aspects of patients’ multiple chronic conditions. Other aspects, such as the impacts on patients’ private lives, family members and friends, are somewhat overlooked [17]. Finally, care management is more difficult when multiple healthcare professionals are involved, which is often the case for patients with multi-morbidity. The quality of a patient’s experience of communication among his/her healthcare professionals deteriorates with an increasing number of conditions [18], and poor communication may contribute to the fragmentation of care [15]. To improve outcomes for patients with multi-morbidity, these complexities and uncertainties concerning the management of care delivery must be adequately addressed and minimised.

Co-creation of care may be valuable for the improvement of care delivery to patients with multi-morbidity [19,20,21,22], as it is especially suitable for situations involving complexity, uncertainty and time constraints [23]. Co-creation of care is based on high-quality and mutually reinforcing communication and relationships between healthcare professionals and patients. In the context of co-creation of care, good communication is characterised as timely, accurate, frequent and problem solving, and good relationships are characterised by shared knowledge, shared goals and mutual respect [23,24]. Frequent (follow-up) meetings between healthcare professionals and patients, at which accurate information is communicated, increase the likelihood of information sharing on personal and medical levels (shared knowledge). Although the sharing of personal information is very important for the setting of treatment goals and alignment of treatment to patients’ wants and needs, it does not always occur [25]. This situation represents a missed opportunity, as not all treatment goals need to be related only to medical aspects, or to all of a patient’s conditions. Moreover, given perceived time constraints, time does not have to be spent on goals that are not important to the patient. Improved healthcare professional–patient communication and relationships also increase patients’ treatment adherence [26,27], and thereby outcomes. In addition, many patients who report good communication and relationships with their healthcare professionals are more satisfied with care [26,28,29] and perceive higher levels of well-being [30]. Many studies of well-being, however, have not distinguished social and physical well-being. As patients with multi-morbidity encounter not only the physical consequences (clinical aspects) of their diseases but also social consequences due to, for example, coping problems and the impacts of their diseases on their personal lives and loved ones, social and physical well-being were examined separately in the present study to enable an understanding of which aspects of well-being are associated with co-creation of care. As overall well-being can be seen as the joint production of social and physical well-being [31], an understanding of the influence of co-creation of care on both well-being domains may contribute to the improvement of care for patients with multi-morbidity through better alignment with patients’ needs.

To our knowledge, only one study involving patients with multi-morbidity in the primary care setting has revealed positive relationships of co-creation of care with satisfaction with care and (social and physical) well-being, using a cross-sectional design [21]. The present study adds to that knowledge by using a longitudinal design. Insight on long-term outcomes and whether improvements in co-creation of care over time are also associated with satisfaction with care and physical and social well-being could aid the improvement of healthcare for this patient population. This is the first longitudinal study investigating the relationships of co-creation of care with physical and social well-being and satisfaction with care among patients with multi-morbidity in the primary care setting.

## 2. Materials and Methods

### 2.1. Participants and Procedure

This study included patients with multi-morbidity (two or more registered chronic conditions, that is asthma, diabetes, COPD, heart and vascular diseases) from seven primary care practices in Noord-Brabant, the Netherlands. These practices are part of a cooperative of GPs from 160 primary care practices in the Netherlands called “Zorggroep RCH Midden Brabant BV”. They were selected because they were considered to be the best practices in this cooperative and because they expressed enthusiasm about further improvement. The participating GP practices identified all eligible patients and provided us with their names and addresses.

In 2017 (T0), a questionnaire was sent to study participants by mail. Those who did not respond after 3 weeks received reminders by mail. Another 3 weeks later, a second reminder and another copy of the questionnaire were sent by mail to the remaining non-responders. Thereafter, a reminder by telephone was given to non-responders for whom telephone numbers were known. Of 413 potential study participants, 19 patients were not eligible to participate due to incorrect addresses (*n* = 5), death (*n* = 4), poor eyesight (*n* = 3), terminal illness (*n* = 2), recent relocation (*n* = 2), inability to fill in the questionnaire due to poor cognitive function (*n* = 2) and recent stroke (*n* = 1), as reported by the patients, their GPs and/or informal caregivers. Of the remaining 394 participants, 216 completed the questionnaire at T0 (55% response rate).

Between T0 and T1, 59 participants dropped out (as reported by the primary care practices) due to death, nursing home or hospice admission, inability to fill in the questionnaire due to poor cognitive function and no longer being treated at the primary care practices. In 2018 (T1), 335 questionnaires were sent to the remaining participants. As at T0, reminders were sent to non-responders after 3 and 6 weeks. Again, 19 patients were not eligible to participate due to incorrect addresses (*n* = 5), death (*n* = 5), poor cognitive function/dementia (*n* = 5), nursing home admission (*n* = 2) and inability to fill in the questionnaire (*n* = 2). Of the remaining 315 participants, 169 completed the questionnaire at T1 (54% response rate). Overall, 138 participants filled in the questionnaires at both T0 and T1; thus, the attrition rate was 36%. A sample size calculation revealed that 124 participants would be required in order to detect small to medium effects with 80% power and a type 1 error rate of 5%. Having 138 respondents is therefore sufficient for valid results.

The medical ethics committee of Erasmus Medical Centre, Rotterdam, the Netherlands, approved the research proposal for this study (file no. METC_2018_021). The committee determined that the rules imposed by the Dutch Medical Research Involving Human Subjects Act did not apply. Written informed consent was obtained from all participants.

### 2.2. Study Design and Setting

This study was part of a larger longitudinal study in which healthcare professionals from the seven primary care practices in Noord-Brabant, the Netherlands, participated in five “knowledge” workshops and four “get togethers” during a year, with the aim of motivating them to deliver more patient-centred care and improve co-creation of care. The knowledge workshops provided information about and training in several interventions (Box 1). These interventions were implemented in the primary care practices. Furthermore, most healthcare professionals made videos of one of their consultations, which were later discussed with a trainer to determine how they could improve their patient-centredness and co-creation of care. All healthcare professionals and the researchers involved in the larger longitudinal study attended the “get togethers”, during which experiences with the interventions and preliminary research results were shared and validated.

Box 1 **Interventions used most frequently in participating primary care practices.**

**Intervention**

**Explanation**
Coaching on the jobDuring two workshops, a coach helped all healthcare professionals employed at two practices improve their patient-centredness. All daily care activities, from appointment making via internet/telephone to front desk work, provision of advice and consultation structure, were evaluated, and required points of improvement were discussed. Shared decision makingDuring one workshop, professionals were trained to use shared decision making during consultations to (1) prepare patients for the decision-making process (e.g., by informing them of consultation goals), (2) determine goals (e.g., jointly explore patients’ situations, share relevant medical information and formulate goals), (3) agree on action points (e.g., by discussing all options) and (4) act and evaluate (e.g., by acting on agreements and reflecting on progression).Training in illiteracy recognitionThis training focused on how healthcare professionals can recognise illiterate patients and adjust their communication accordingly during consultations, answering of the telephone by triage assistants and at the front desk. For example, the teach-back method can be used to make sure patients understand all information provided, and informational materials can be adjusted. Three good questionsThis intervention is based on a Dutch national campaign that aims to reassure patients that their wishes, anxieties and needs matter during healthcare consultations. The three good questions that patients can ask their healthcare professionals are (1) What are my options? (2) What are the pros and cons of those options? and (3) What does that mean in my situation? To make patients more aware of their role during consultations, the practices provided fliers with the three questions at the front desk and in the waiting room and showed the questions on a screen in the waiting room.Motivational interviewingTraining in a directive, patient-centred approach to counselling that prepares patients for behaviour changes and helps to resolve ambivalence. Diary keepingAll healthcare professionals kept diaries on how they improved/changed their care delivery during the year (e.g., listening to the patient for 1 min at the beginning of a consultation before talking, making sure the patient’s question is the central starting point of the consultation and not judging or interpreting the patient’s feelings without asking.

### 2.3. Measures

#### 2.3.1. Background Characteristics

Patients were asked to provide information on their background characteristics, such as age, gender, education and marital status. We dichotomised marital status (1, living alone, widowed or divorced; 0, married/living with partner) and education (1, primary education or less; 0, preparatory school for vocational secondary education or higher).

#### 2.3.2. Co-creation of Care

Following previous research, we used the relational co-production instrument to assess co-creation of care [23]. This instrument of 7 items is used to evaluate aspects of communication (whether it is timely, accurate, frequent and problem solving), and the healthcare professional–patient relationship (shared goals, shared knowledge and mutual respect). Example questions are “How often do you communicate with your GP/nurse practitioner/specialist?” and “To what extent do these people (GP/nurse practitioner/specialist) share your goals?” Responses are given on a scale ranging from 1 (never) to 5 (always), with higher mean scores representing better co-creation of care. In this study, Cronbach’s alpha values for the relational co-production instrument at T0 and T1 were 0.93 and 0.96, respectively, indicating good reliability. Change in co-creation of care was measured by subtracting the mean score at T0 from that at T1.

#### 2.3.3. Well-being

Well-being was assessed at T0 and T1 using the 15-item version of the Social Production Function Instrument for the Level of Well-being short (SPF-ILs) [32]. This instrument is used to measure levels of physical (comfort and stimulation) and social (behavioural confirmation, affection and status) well-being. Example questions are “Do you feel that people really love you?” and “Are your activities challenging to you?” Responses are given on a scale ranging from 1 (never) to 4 (always), with higher mean scores representing greater well-being. In this study, Cronbach’s alpha values for the SPF-ILs at T0 and T1 were 0.88 and 0.87, respectively, indicating good reliability.

#### 2.3.4. Satisfaction with Care

Satisfaction with care was measured using the 6-item version of the Satisfaction with Stroke Care (SASC) questionnaire [33]. The use of this instrument is not restricted to stroke patients, as it contains general questions about satisfaction with care; the SASC questionnaire has been used for various patient populations in the hospital setting [34,35], and adjusted versions have been used in other care settings [21,36]. Example items are “The staff has done everything they can to make me well again”, “I am happy about the effect treatments had on my disease progression” and “I have received all the information I want about the causes and nature of my illness(es)”. Responses are given on a scale ranging from 1 (totally disagree) to 4 (totally agree), with higher mean scores representing greater satisfaction with care. In this study, Cronbach’s alpha values for the SASC instrument at T0 and T1 were 0.87 and 0.92, respectively, indicating good reliability.

### 2.4. Statistical Aanalyses

We used SPSS software (version 23; IBM Corporation, Armonk, NY, USA) to analyse the data. First, we calculated descriptive statistics [frequencies, percentages, means, ranges and/or standard deviations (SDs)] for all variables to characterise the study population. Second, paired-sample *t* tests were used to investigate improvements over time (differences between T0 and T1) in co-creation of care, physical and social well-being and satisfaction with care. Third, regression analyses were performed to investigate multivariate relationships among these variables. As age, gender, marital status and education are known to be related to well-being and satisfaction with care, we controlled for these variables in the multivariate regression analysis [37,38,39,40]. Results were considered to be significant when two-sided *p* values were ≤0.05.

Because some data on aspects underlying co-creation of care were missing, we performed additional regression analyses with imputed data produced with the Markov Chain monte Carlo imputation technique (*n* = 138). As these analyses yielded similar results, only the results of the original analyses are presented in the tables. Furthermore, we checked for multilevel nesting within the GP practices. We found no variance at the GP practice level (data available on reasonable request), indicating that nesting did not affect our conclusions.

## 3. Results

Table 1 presents an overview of the background characteristics of the 138 patients with multi-morbidity who filled in questionnaires at both T0 and T1. The mean age of the respondents at T1 was 73.50 (range 48.45–94.32, SD = 9.99) years; 42.2% of respondents were male, 37.2% were single and 33.8% had low educational levels. The mean scores for co-creation of care and satisfaction with care were 3.86 ± 0.80 and 3.20 ± 0.43, respectively, and those for social and physical well-being were 2.90 ± 0.47 and 2.80 ± 0.55, respectively.

Co-creation of care improved significantly over time (*t* = 2.25, *p* = 0.026; Table 2). To better understand this improvement, we also performed paired-sample *t* tests for individual aspects underlying co-creation of care. All aspects underlying co-creation of care improved over time, although only two improvements were significant: frequent communication (*t* = 2.94, *p* = 0.004) and timely communication (*t* = 2.51, *p* = 0.013). Mean scores for these two aspects were lower than those of the other aspects at T0 (3.2 and 3.51, respectively). In Table 3, the results of the paired sample *t* tests of the dependent variables (social well-being, physical well-being, and satisfaction with care) are presented. Improvement over time was also observed for social well-being (*t* = 2.31, *p* = 0.022) and physical well-being (*t* = 2.72, *p* = 0.007) but not for satisfaction with care (*t* = 0.18, *p* = 0.858).

The results of the multivariate regression analysis are presented in Table 4. Improvement in co-creation of care over time (T1–T0) was related significantly to social well-being (*β* = 0.288, *p* = 0.002). The inclusion of background characteristics, social well-being at baseline, co-creation of care at baseline and the change in co-creation of care over time explained 42.6% of the variance in social well-being (*r*^2^ = 0.426, *F* = 11.255). Improvement in co-creation of care was also related significantly to physical well-being (*β* = 0.345, *p* ≤ 0.000). The inclusion of background characteristics, physical well-being at baseline, co-creation of care at baseline and the change in co-creation of care over time explained 44.5% of the variance in physical well-being (*r*^2^ = 0.445, *F* = 12.470). Co-creation of care at baseline and improvement therein were related significantly to satisfaction with care (*β* = 0.326, *p* = 0.008 and *β* = 0.501, *p* ≤ 0.000, respectively). The inclusion of background characteristics, satisfaction with care at baseline, co-creation of care at baseline and changes in co-creation of care over time explained 19.5% of the variance in satisfaction with care (*r*^2^ = 0.195, *F* = 3.603).

## 4. Discussion

This study was the first to investigate longitudinal relationships between co-creation of care, physical well-being, social well-being and satisfaction with care among patients with multi-morbidity in the primary care setting in Noord-Brabant, the Netherlands. Our findings clearly show that improvements in co-creation of care, as perceived by patients with multi-morbidity, benefit these patients’ physical well-being, social well-being and satisfaction with care, highlighting the value of investment in co-creation of care in the primary care setting.

Patients participating in this study perceived that co-creation of care improved significantly over time, likely due to the GP practices’ investment during the 1-year study period. For example, the shared decision-making intervention likely contributed to the establishment of shared goals, the “three good questions” intervention likely contributed to the generation of shared knowledge, and the illiteracy training likely improved communication between healthcare professionals and patients. The findings of this study do not, however, provide insight into which specific interventions contributed to the improvements in co-creation of care; further research is recommended to identify interventions that most effectively improve the co-creation of primary care, and the reasons for this effectiveness. Nevertheless, given the variation among patients with multi-morbidity in their needs for support and care [41] goals and the need to co-create, we emphasise the need to invest in a variety of interventions to make sure that the co-creation of care is well adjusted and personalised for all patients.

Although this study revealed overall improvement in co-creation of care, only frequent and timely communication improved significantly over time. The primary care practices that participated in this study are among the best-performing practices in their region (they were selected for this reason), which is reflected in the high baseline scores for co-creation of care. Thus, the ceiling effect may explain the non-significant improvement in some underlying elements. Scores for frequent and timely communication were lower than those for other aspects and thus may have been easier to improve. Examination of the effects of investment in co-creation of care by average- and/or low-scoring GP practices would be of interest. We expect that the effects of investment in co-creation of care would be greater in average- and low-scoring GP practices, where improvement would be easier to achieve. However, motivating these practices to invest in co-creation of care would probably be more difficult than for the practices included in this study.

The social and physical well-being of patients with multi-morbidity also improved over time, and changes in co-creation of care contributed to this improvement. Co-creation of care at baseline and changes therein were related to patients’ satisfaction with care, but the mean satisfaction with care score did not improve significantly over time. Although there was no improvement in satisfaction with care over time based on the group mean, the change in co-creation of care may have caused individual variation in satisfaction with care, which may explain the significant longitudinal relationship found.

Improvement in co-creation of care also showed significant longitudinal relationships with the social and physical well-being of patients with multi-morbidity. These findings are in accordance with our expectations and in partial agreement with cross-sectional data showing that co-creation of care was related to the social well-being and satisfaction with care (but not physical well-being) of patients with multi-morbidity in a primary care setting [21]. The discrepancy in the physical well-being findings may be explained by the improbability that the main elements of co-creation of care (communication and relationship quality) immediately enable the realisation of physical well-being goals; they may, however, have a cumulative effect in the long term [42]. Street and colleagues [39] also suggested that communication can lead to improved physical health when conversations improve the understanding of patients’ conditions (e.g., enable correct diagnoses) and better alignment of treatments to patients’ situations and conditions. The findings of this study reinforce the need for GP practices to continue to invest in co-creation of care to improve physical well-being, as well as social well-being and satisfaction with care, among patients with multi-morbidity.

This study has several limitations that should be considered. First, as it was conducted in Noord-Brabant, the Netherlands, the generalisability of our findings may be limited; further research in other countries and/or regions is recommended. Second, each chronic condition, and combinations thereof, may have affected the study outcomes. We lacked information about individual participants’ conditions, aside from the presence of some combination of asthma, diabetes, COPD and heart and vascular diseases, as the GP practices were not allowed to share this information due to privacy concerns. Third, we do not have information on drug therapy or activities of daily living, which may have an influence on our study outcomes. Fourth, only patients who filled in the questionnaire at both T0 and T1 were included in this study; 36% (*n* = 78) of patients filled in the questionnaire only at baseline and were excluded. The attrition rate could be considered a limitation to this study. Attrition rates tend to be associated positively with increased age, poor functioning, cognitive impairment and unmarried status [43]. Patients with multi-morbidity constitute a vulnerable population, which could explain the high attrition rate. This dropout may have affected our findings, given the existence of significant differences in health and well-being between patients who dropped out and the remaining sample: at baseline, those who dropped out were significantly older and lower educated, significantly more of them were single, and they had significantly lower scores for physical and social well-being, satisfaction with care and co-creation of care. The more favourable evaluation of co-creation of care by the remaining sample may have caused underestimation of improvement in co-creation of care, as improvement could have been greater in the total sample.

## 5. Conclusions

In this study, improvement in co-creation of care was related positively to the physical and social well-being and satisfaction with care of patients with multi-morbidity in primary care. The findings of this study are important because the management of care delivery to this patient population is often considered to be complex. They indicate the value of investment in co-creation of care to improve outcomes for patients with multi-morbidity in the primary care setting.

## Figures and Tables

**Table 1:** Descriptive statistics at T1.

	Mean ± Standard Deviation (Range/Absolute Number) or Percentage
Age (years)	73.50 ± 9.99 (48.45–94.32)
Gender (male)	42.2% (57)
Marital status (single)	37.2% (51)
Education level (low)	33.8% (46)
Satisfaction with care	3.20 ± 0.43 (2–4)
Social well-being	2.90 ± 0.47 (1.56–3.78)
Physical well-being	2.80 ± 0.55 (1–4)
Co-creation of care	3.86 ± 0.80 (1–5)

*Note*. The analysis included only data from respondents who filled in questionnaires at both T0 and T1 (*n* = 138).

**Table 2:** Paired sample *t* tests (aspects of) co-creation of care.

		T0		T1		Paired Difference		
Variable	*n*	Mean	SD	Mean	SD	*t*	df	*p*
Co-creation of care	135	3.70	0.88	3.87	0.78	2.25	134	0.026
Frequent communication	135	3.20	0.84	3.44	0.87	2.94	134	0.004
Timely communication	132	3.51	1.05	3.75	0.90	2.51	131	0.013
Accurate communication	131	3.86	1.01	4.01	0.86	1.68	130	0.095
Problem-solving communication	124	3.95	1.07	4.10	0.83	1.44	123	0.153
Shared knowledge	121	3.81	1.10	3.91	0.94	0.852	120	0.396
Mutual respect	114	3.95	1.06	4.12	0.88	1.54	113	0.127
Shared goals	116	3.92	1.05	4.06	0.94	1.38	115	0.171

**Table 3:** Paired sample *t* tests social well-being, physical well-being and satisfaction with care.

		T0		T1		Paired Difference		
Variable	*n*	Mean	SD	Mean	SD	*t*	df	*p*
Social well-being	132	2.80	0.50	2.90	0.47	2.31	131	0.022
Physical well-being	135	2.67	0.57	2.79	0.55	2.72	134	0.007
Satisfaction with care	125	3.19	0.50	3.20	0.42	0.18	124	0.858

**Table 4:** Multivariate relationships between co-creation of care, satisfaction with care, social and physical well-being over time.

	Social Well-Being				Physical Well-Being				Satisfaction with Care			
	*B*	SE	*β*	*p*	*B*	SE	*β*	*p*	*B*	SE	*β*	*p*
(Constant)	1.395	0.378		0.000	1.788	0.421		0.000	2.603	0.424		0.000
Outcome at T0 *	0.593	0.074	0.617	0.000	0.584	0.071	0.612	0.000	0.054	0.088	0.060	0.544
Age	0.007	0.004	0.153	0.054	0.009	0.004	0.169	0.030	0.004	0.004	0.096	0.307
Gender	0.017	0.077	0.017	0.825	0.063	0.86	0.57	0.465	0.064	0.081	0.074	0.434
Marital status	0.105	0.080	0.106	0.191	0.047	0.090	0.041	0.604	0.021	0.085	0.024	0.802
Education	0.215	0.080	0.210	0.008	0.149	0.090	0.126	0.102	0.129	0.086	0.142	0.136
Co-creation of care	0.065	0.050	0.120	0.202	0.013	0.056	0.021	0.818	0.165	0.062	0.326	0.008
Change in co-creation of care over time	0.157	0.050	0.288	0.002	0.216	0.56	0.345	0.000	0.240	0.054	0.501	0.000

* “Outcome” refers to satisfaction with care, social well-being and physical well-being.
